# The stress response factor *daf-16*/FOXO is required for multiple compound families to prolong the function of neurons with Huntington’s disease

**DOI:** 10.1038/s41598-017-04256-w

**Published:** 2017-06-21

**Authors:** Francesca Farina, Emmanuel Lambert, Lucie Commeau, François-Xavier Lejeune, Nathalie Roudier, Cosima Fonte, J. Alex Parker, Jacques Boddaert, Marc Verny, Etienne-Emile Baulieu, Christian Neri

**Affiliations:** 1 CNRS, Laboratory of Neuronal Cell Biology & Pathology and University Hospital Department Fight Aging and Stress (DHU FAST), UMR 8256 Paris, France; 2 Sorbonne Universités, University Pierre and Marie Curie (UPMC) Univ Paris 06, Paris, France; 3 Inserm, UMR 1195, 94276 Le Kremlin-Bicêtre, Cedex, France; 4 Department of Geriatrics, Pitié-Salpêtrière Hospital, Assistance Publique Hôpitaux de Paris (APHP), 75013 Paris, France; 5 MAPREG, 94276 Le Kremlin-Bicêtre, Cedex, France; 6 CRCHUM, Montréal, Canada and Department de Neurosciences, Faculté de médecine, Université de Montréal, Montréal, Canada

## Abstract

Helping neurons to compensate for proteotoxic stress and maintain function over time (neuronal compensation) has therapeutic potential in aging and neurodegenerative disease. The stress response factor FOXO3 is neuroprotective in models of Huntington’s disease (HD), Parkinson’s disease and motor-neuron diseases. Neuroprotective compounds acting in a FOXO-dependent manner could thus constitute *bona fide* drugs for promoting neuronal compensation. However, whether FOXO-dependent neuroprotection is a common feature of several compound families remains unknown. Using drug screening in *C. elegans* nematodes with neuronal expression of human exon-1 huntingtin (128Q), we found that 3ß-Methoxy-Pregnenolone (MAP4343), 17ß-oestradiol (17ßE2) and 12 flavonoids including isoquercitrin promote neuronal function in 128Q nematodes. MAP4343, 17ßE2 and isoquercitrin also promote stress resistance in mutant *Htt* striatal cells derived from knock-in HD mice. Interestingly, *daf-16*/FOXO is required for MAP4343, 17ßE2 and isoquercitrin to sustain neuronal function in 128Q nematodes. This similarly applies to the GSK3 inhibitor lithium chloride (LiCl) and, as previously described, to resveratrol and the AMPK activator metformin. Daf-16/FOXO and the targets engaged by these compounds define a sub-network enriched for stress-response and neuronally-active pathways. Collectively, these data highlights the dependence on a *daf-16*/FOXO-interaction network as a common feature of several compound families for prolonging neuronal function in HD.

## Introduction

Key lifespan modulators such as the FOXO (Forkhead box O), AMPK (AMP-activated protein kinase) and sirtuin (Sir2) proteins are central regulator of cellular homeostasis and survival mechanisms in response to stress^1, 2^. As such, this class of proteins is believed to constitute an interesting target space for sustaining the capacity of neurons to compensate for proteotoxic stress and maintain function in neurodegenerative diseases (neuronal compensation)^3^. Neuronal compensation is a biological phenomenon that may be particularly relevant to slowing-down the early phases of neurodegenerative disease processes, *e.g*. before neurons face unmanageable stress and cell death^4–11^. Drugs able to promote neuronal compensation mechanisms^12^ could thus enhance the resistance of the brain to neurodegenerative diseases. Interestingly, steroids can modulate longevity in *Caenorhabditis elegans* (*C. elegans*) through lifespan modulators such as *daf-12*, a nuclear receptor that regulates the dauer diapause and developmental age^13^. Additionally, steroid ligands (dafachronic acids) may bind and transactivate DAF-12^14^ and regulate aging^15^. Here, we hypothesized that neurosteroids may promote the function of vulnerable neurons in neurodegenerative diseases and that neuroprotection by these compounds may involve stress response and cell survival pathways. We also hypothesized this may represent a common feature of several families of neuroprotective compounds. To test for this hypothesis, we used a *C. elegans* model of neuronal dysfunction in Huntington’s Disease (HD)^16^. In this model, transgenic nematodes with stable expression of human polyglutamine (polyQ)-expanded (128Q) exon-1 huntingtin (HTT) fused to fluorescent proteins in touch receptor neurons show defective posterior mechanosensation, a progressive phenotype that is detectable in L4 larvae with aggravation in young adult animals and that is accompanied by axonal dystrophy^16, 17^. These nematodes provide a genetically- and pharmacologically-tractable system to manipulate the neuronal dysfunction induced by polyQ-expanded HTT species *in vivo* ^4^. The use of these nematodes revealed that the capacity of the stress-response transcription factor FOXO3 to protect neurons from the cytotoxicity of mutant *HTT* ^6^ is altered by abnormal neurodevelopmental signalling during the early phases of the HD pathology, before symptoms^18^. This is caused by increased levels of the axon guidance and Wnt receptor Ryk, which translates into elevated nuclear levels of the intracellular fragment of Ryk (Ryk-ICD), altering the ability of the ß-catenin-FOXO3 complex to regulate gene transcription^18^. This suggests that pharmacological manipulation of pathways that normally converge onto FOXO3 to regulate stress response may restore the homeostasis of the ß-catenin-FOXO3 complex and promote neuronal compensation in HD. Here, we report that steroids 3ß-Methoxy-Pregnenolone (MAP4343)^19^ and 17ß-oestradiol (17ßE2) promote neuronal function in 128Q nematodes, an effect that requires *daf-16*/FOXO. Additionally, MAP4343 and 17ßE2 reduces stress vulnerability in mutant *Htt* mouse striatal cells derived from HdhQ111 knock-in mice^20^, protecting them against pyknosis as they undergo cell death upon serum deprivation. We also report *daf-16*/FOXO-dependent protection of neuronal function by flavonoid analogs with a resveratrol-like pharmacophore such as isoquercitrin^21^ and by GSK-3 inhibitors with neuroprotective effects in HD mice such as LiCl^22^. Interestingly, neuroprotection in 128Q nematodes is also dependent on *daf-16*/FOXO interactors that may be individually used by these compounds as biological targets, including *ptl-1*/MAP for MAP4343, *daf-12/*NR1H3 for 17ßE2, *sir 2.1*/SIRT1 (putative target) for isoquercitrin and *bar-1*/ß-catenin for LiCl (via GSK3). Together with *daf-16*/FOXO, these targets define a Wormnet sub-network that has higher-than-expected connectivity and that is enriched for stress-response pathways and neuronally-expressed genes. These results suggest that dependence on a *daf-16*/FOXO-interaction network is a common feature of multiple compound families such as neurosteroids, flavonoid analogs and GSK-3 inhibitors. Together with our previously-described results on the *daf-16*/FOXO-dependent protection by metformin, an AMPK activator, against neuronal dysfunction in 128Q nematodes^23^, these results document the therapeutic potential of several compound families for promoting neuronal compensation in HD via stress response networks.

## Results

### 3ß-methoxypregnelonone and 17ß-oestradiol promote neuronal function in 128Q nematodes in a *daf-16*/FOXO-dependent manner

**Figure 1:**
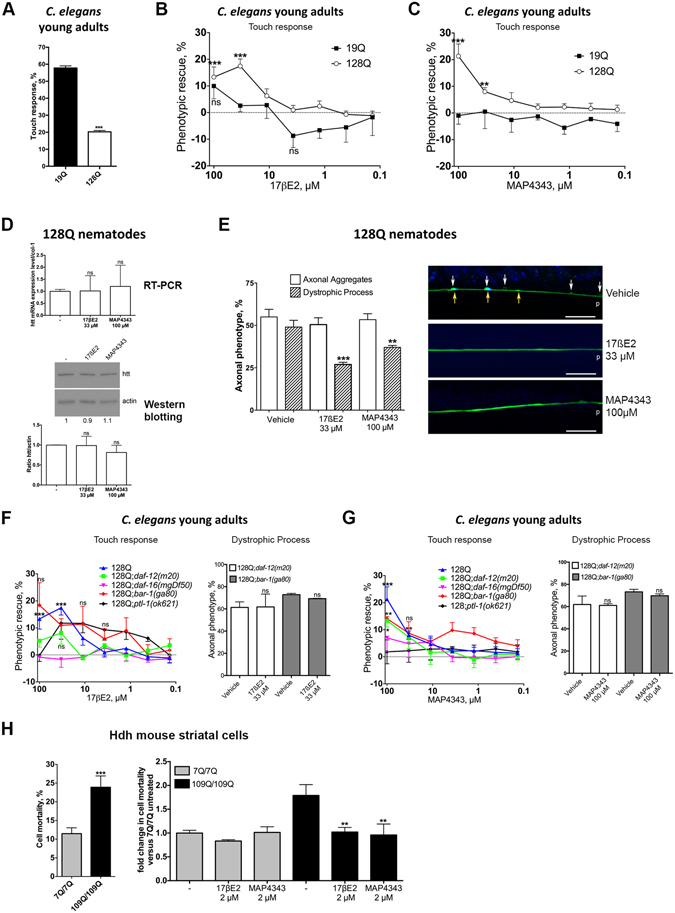
17βE2 and MAP4343 protect nematode neurons and mouse striatal cells from expanded polyQ cytotoxicity. Nematode data are mean ± SEM for > 200 nematodes in each group as tested in a total of at least 4 independent experiments. The percentage of phenotypic (touch sensitivity) rescue is calculated as ((test − control)/(100 − control)*100). The maximally achievable level of phenotypic rescue in 128Q nematodes (touch response level back to that of 19Q nematodes) is 35%. A negative value for phenotypic rescue means aggravation of tail Mec phenotype. (**A**) 128Q nematodes show a strong reduction of touch response compared to 19Q nematodes (***P < 0.001), a progressive phenotype detectable in L4 larvae and becoming severe in young adults^4^. (**B**) The most effective concentration for 17βE2 neuroprotection is 33 μM (***P < 0.001). ns: not significant. (**C**) The most effective concentration for MAP4343 neuroprotection is 100 μM (**P < 0.01 and ***P < 0.001). (**D**) 17βE2 and MAP4343 do not alter transgenic RNA and protein levels in 128Q animals. A cropped representative western blot is shown. The corresponding full-length blot is shown in Fig. S4. (**E**) 17βE2 and MAP4343 reduce axonal dystrophy in 128Q nematodes (*P < 0.05) with no effect detected on axonal aggregates. The right panel shows a representative image of the anterior process of posterior touch receptor neurons of 128Q nematodes co-expressing HTT1-57::CFP and YFP. Swelling (white arrows, YFP signals are pseudocolored in green) and HTT::CFP aggregation (yellow arrows, CFP signals are pseudocolored in blue) are shown. p: posterior end. Magnification is 100 × and scale bar is 10 µM. (**F**) The left panel shows that neuroprotection by 17ßE2 (100 µM and 33.3 µM) is primarily dependent on *daf-16*/FOXO and its interactor *daf-12*, which may also involve *bar-1*. The *P* values are shown only for phenotypic rescue in 128Q nematodes to ensure readability. ***P < 0.001 versus 128Q animals treated with vehicle. ns: not significant (red, black and green curves). The percentage of phenotypic rescue at the most effective drug concentration (Rmax) is indicated in Table 2. The right panel shows that 17ßE2 protection against axonal dystrophy is lost in 128Q nematodes with loss-of-function of *daf-12* or *bar-1*. (**G**) The left panel shows that neuroprotection by MAP4343 (100 µM and 33.3 µM) is primarily dependent on *daf-16*/FOXO and *ptl-1*/MAP (see Table 2 for Rmax). The *P* values are shown only for phenotypic rescue in 128Q nematodes. *P < 0.05 (green curve), **P < 0.01 (red and blue curves) and ***P < 0.001 versus 128Q nematodes treated with vehicle. ns: not significant (red and green curves). The right panel shows that MAP4343 protection against axonal dystrophy is lost in 128Q nematodes with loss-of-function of *daf-12* or *bar-1*. (**H**) The vulnerability to cell death of mutant *Htt* mouse striatal cells is reduced by 17βE2 and MAP4343. Data are mean ± SD for > 150 cells in each group as tested in at least three independent experiments (**P < 0.01). HTT levels are unchanged by drug treatment (Fig. S1).

Integrated *C. elegans* transgenics co-expressing exon 1 HTT with 128 glutamines and YFP in touch receptor neurons (strain ID1) have consistently shown a stable behavior over time in which young adults show a dramatic loss of touch response at the tail (20–22% response) compared to control animals (strain ID245) co-expressing exon 1 HTT with 19 glutamines (19Q) and YFP (Fig. 1A), a phenotype accompanied by axonal dystrophy^4, 6, 18, 23, 24^. In these animals, defective mechanosensation is dependent on the *HTT* transgene as transgenic animals expressing YFP alone retain a high level (77–79%) of response to light touch at the tail^17^. Using 128Q nematodes, we tested nine substances including hormonal pregnenolone, the pregnenolone derivative MAP4343^25^, 17ßE2, 17α-oestradiol, 2-Methoxy-oestradiol, testosterone, dehydroepiandrosterone (DHEA), 3βmethyl-Δ5androstene-17one (3Me-D5A) and the immunosuppressor FK506 (Table 1). Among these nine substances, only 17βE2 and MAP4343 protect from the loss of response to light touch in 128Q nematodes (Fig. 1B,C), with no activity detected in 19Q nematodes (Fig. 1B,C) and no decrease detected in transgenic mRNA and protein levels (Fig. 1D). The lack of pregnenolone neuroprotection could relate to the higher metabolic stability of MAP4343 (EEB, personal communication). At the cellular level, 17βE2 and MAP4343 reduce axonal dystrophy in 128Q animals, while axonal HTT aggregation is not altered (Fig. 1E). Thus, 17βE2 and MAP4343 specifically reduce neuronal dysfunction and axonal dystrophy in 128Q nematodes.

**Table 1:** Effect of steroids and FK506 on neuronal dysfunction of 128Q nematodes.

Compound	Effect on 128Q cytotoxicity			Comment
	Rmax, %	EC50, µM	ED50, µM	
17ßE2	17.4 ± 2.7	25	n.a.	Protection
2-MetoxyE2	6.8 ± 3.2	n.a.	n.a.	No protection
17aE2	6.5 ± 7.4	n.a.	n.a.	No protection
MAP4343	21.3 ± 4.6	n.a.	50	Protection
Pregenenolone	2.5 ± 1.8	n.a.	n.a.	No protection
Testosterone	5.4 ± 1.6	n.a.	n.a.	No protection
DHEA	6.3 ± 3.1	n.a.	n.a.	No protection
3Me-D5A	3.1 ± 1.9	n.a.	n.a.	No protection
FK506	13.5 ± 3.1	n.a.	n.a.	No protection

Nematodes were scored for sensitivity to posterior touch at various compound concentrations (100–0.1 µM). The percentage of phenotypic (touch sensitivity) rescue at the most effective compound concentration relative to untreated 128Q nematodes is indicated as the Rmax. The EC50 is the half maximal effective concentration. The Rmax is the percentage of rescue at most effective concentration of drug relative to untreated control. The ED50 is the dose of a drug that is pharmacologically effective for 50% of the population. n.a.: not applicable (see shape of dose-response curve).

Next, we asked whether neuroprotection by 17βE2 and MAP4343 may depend on *daf-16*, the DAF-16 co-factor *bar-1*/ß-catenin^26^ and *daf-12/*NR1H3, the latter which may work upstream to *daf-16* as inferred from the study of diapause^27^. We previously showed that neuronal dysfunction in 128Q nematodes (Fig. 1A) is increased by loss-of-function (LOF) mutants of *daf-16* such as *daf-16(mu86)* and *daf-16(mgDf50)* and LOF mutants of *bar-1* such as *bar-1(ga80)* ^6, 18^. We also showed that neuroprotection by increased Sir2 dosage and by the putative sirtuin-activator resveratrol requires *daf-16*/FOXO in 128Q nematodes^4^. Here, we observed that the neuroprotective effects of 17ßE2 and MAP4343 are lost in 128Q nematodes carrying the null allele *daf-16(mgDf50)* (Fig. 1F,G), a mutation that does not change transgene expression as previously shown by us^4^. In contrast, *bar-1(ga80)*, a LOF allele that does not modify transgene expression^6^, reduces MAP4343 protection against neuronal dysfunction (Fig. 1G, left panel) and suppresses protection against axonal dystrophy (Fig. 1G, right panel; see also Fig. 1E), suggesting MAP4343 protection partially depends on *bar-1*. Regarding 17ßE2, the large variation of phenotypic rescue (Fig. 1F/left panel) and loss of protection againt axonal dystrophy (Fig. 1F/right panel; see also Fig. 1E) suggest that 17ßE2 neuroprotection may be dependent on *bar-1*. In contrast to LOF mutants of *daf-16*/FOXO, LOF mutants of *daf-12* such as *daf-12(m20)* ^13^ and *daf-12(rh61rh411*) do not significantly modify the level of neuronal dysfunction in 128Q nematodes (touch response in 128Q;*daf-12(m20)* nematodes: 21.9 ± 3.4%), suggesting that *daf-12* is dispensable to the response to the 128Q transgene in basal conditions. Nonetheless, neuroprotection by 17βE2 is lost in 128Q;*daf-12(m20)* nematodes (Fig. 1F), suggesting that *daf-12* is required for 17βE2 neuroprotection. MAP4343 protection against neuronal dysfunction is reduced in 128Q;*daf-12(m20)* nematodes (Fig. 1G/left panel) and MAP4343 protection against axonal dystrophy is lost in 128Q;*daf-12(m20)* nematodes (Fig. 1G/right panel). Therefore, 17βE2 neuroprotection depends on *daf-16* and *daf-12*, whereas MAP4343 neuroprotection depends on *daf-16* and partially depends on *daf-12* and *bar-1* (Table 2). To further investigate the differences between the pharmaco-genetic profiles of MAP4343 and 17βE2, we tested for the effects of LOF of the microtubule-associated protein (MAP) *ptl-1*. In mammals, MAP4343 promotes neurite extension *in vitro* by binding to MAP2, acting as a specific receptor involved in microtubule polymerization^19^. The *C. elegans* homolog of *MAP2* is *ptl-1*, which is expressed in mechanosensory neurons^28^. Although LOF mutation *ptl-1(ok621)*, a null allele, does not modify neuronal dysfunction in 128Q nematodes (touch response in 128Q;*ptl-1(ok621)* animals: 23.6 ± 3.2%), it abolishes MAP4343 neuroprotection (Fig. 1G), suggesting that neuroprotection by MAP4343 is dependent on *ptl-1*. In contrast, the LOF mutation *ptl-1(ok621)* does not abolish neuroprotection by 17βE2 (Fig. 1F,G), with an apparent shift of EC50 towards lower concentrations, however at the expense of Rmax. Collectively, these data suggest that while MAP4343 and 17βE2 share dependence on *daf-16* as a common feature for neuroprotection against the 128Q transgene, they might use different pathways that involve either MAPs for response to MAP4343 or nuclear hormone receptors for response to 17ßE2.

**Table 2:** Genetic profile of 17ßE2 and MAP4343 protection against neuronal dysfunction in 128Q nematodes.

Genotype	17ßE2 (33 µM)		MAP4343 (33 µM)	
	Rmax, %	Loss of protection	Rmax, %	Loss of protection
128Q	17.4 ± 2.7	—	21.3 ± 4.6	—
19Q	2.6 ± 2.3	—	0.5 ± 6.4	—
*128Q;daf-16(mgDf50)*	2.6 ± 2.9	yes	7.0 ± 5.1	yes
*128Q;daf-12(m20)*	8.1 ± 4.2	yes	13.3 ± 2.3	partial
*128Q;bar-1(ga80)*	no conclusion	no conclusion	14.1 ± 0.8	partial
*128Q;ptl-1(ok621)*	11.7 ± 5.9	no	2.9 ± 0.8	yes

Percent rescue of touch sensitivity by 17ßE2 and MAP4343 is calculated relative to untreated 128Q nematodes at the most effective drug concentration (Rmax). The corresponding dose-response curves are shown in Fig. 1F,G. Partial loss of protection refers to a decrease of protection for which a statistically significant Rmax effect is retained compared to untreated animals.

### 17ß-oestradiol and 3ß-methoxypregnelonone protect mutant *Htt* mouse striatal cells from stress vulnerability

Models based on expression of N-terminal HTT such as exon 1 HTT may not completely recapitulate HD pathogenesis^29, 30^. We thus investigated steroid activity in a model reflecting a closer situation to HD patients, *i.e*. striatal cells derived from the striatum of HdhQ111 knock-in mice^20^. In this cellular model, mutant *Htt* striatal cells are highly sensitive to cell death induced by serum deprivation (stress vulnerability) compared to normal *Htt* cells, a phenotype that is mostly dependent on mutant *Htt* expression^6^. 17βE2 and MAP4343 decrease stress vulnerability of mutant (109Q/109Q) *Htt* cells, with no effect detected in normal (7Q/7Q) *Htt* cells (Fig. 1H). This effect does not involve a reduction of Htt expression (Fig. S1). Thus, 17βE2 and MAP4343 may protect from the stress vulnerability induced by full length Htt expression in HD mouse striatal cells.

### *daf-16*/FOXO-dependent protection of neuronal function in 128Q nematodes is a common feature of several compound families

**Figure 2:**
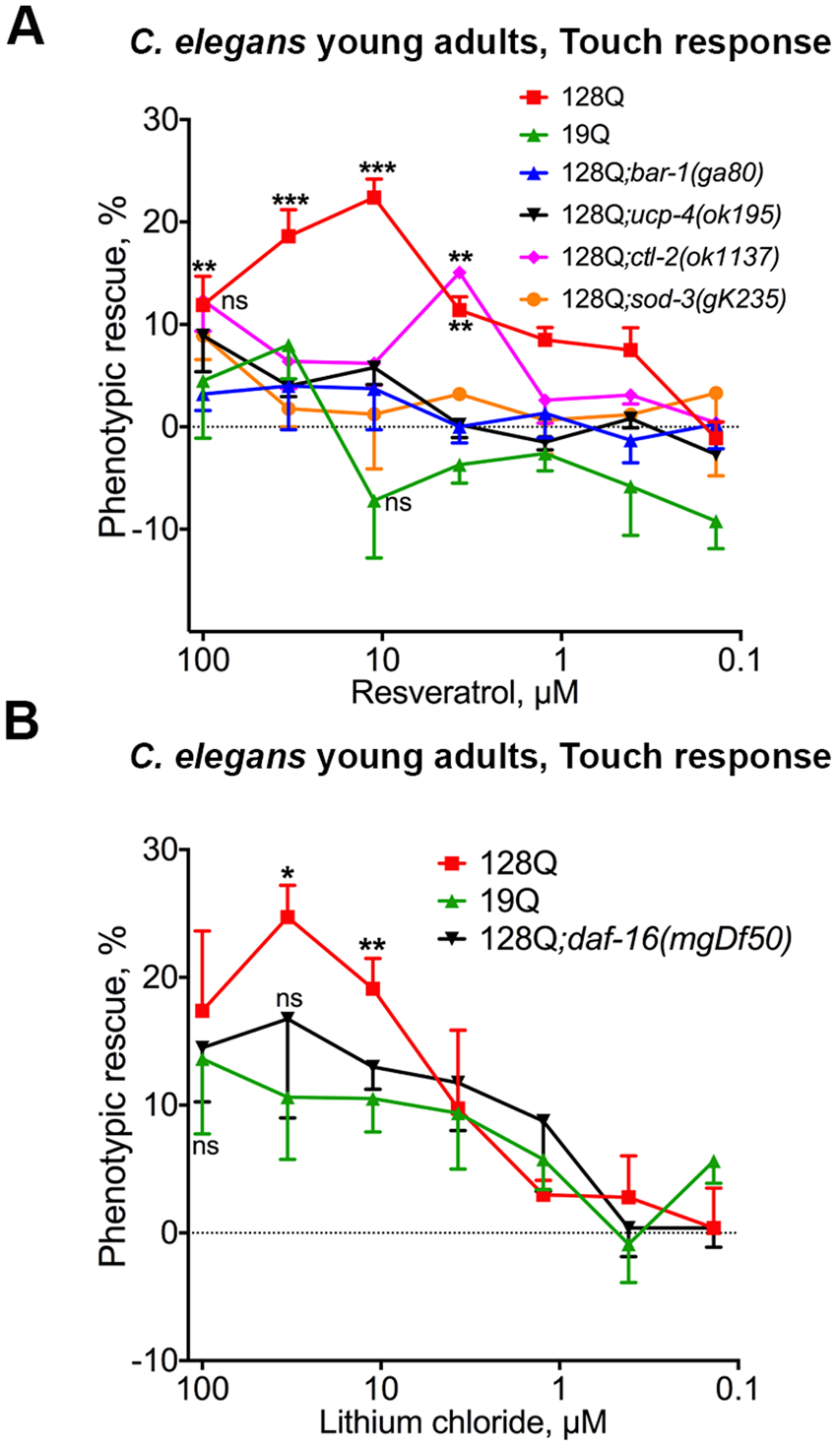
Genetic profiles for neuroprotection by resveratrol and LiCl in 128Q nematodes. Data are mean ± SEM for > 200 nematodes in each group and a total of at least 4 independent experiments in all panels. ns: not significant. (**A**) Resveratrol is dependent on *bar-1*/ß-catenin, *ucp-4*/UCP and antioxidant enzymes for neuroprotection in *C. elegans*. Resveratrol protection is lost in *bar-1*/ß-catenin, *ucp-4*/UCP, *ctl-2*/catalase and *sod-*3/superoxide dismutase mutants. **P < 0.01 and ***P < 0.001 *versus* DMSO-treated 128Q animals. (**B**) LiCl sustains the function of 128Q nematode neurons in a manner that is partially dependent on *daf-16*/FOXO, with no effects detected in 19Q nematodes. *P < 0.05 and **P < 0.01 *versus* DMSO-treated 128Q animals.

The dependence on *daf-16*/FOXO for 17βE2 and MAP4343 to protect 128 nematodes from neuronal dysfunction (Fig. 1) is also true for resveratrol, a flavonoid that is protective in *C. elegans* and mouse cell models of HD pathogenesis^4^. While resveratrol may have biological properties with therapeutic potential in several diseases^31, 32^, there was a debate on the targets that may be engaged by resveratrol and, more largely, by sirtuin-activating compounds (STACs) to have a beneficial effect on lifespan^33^ or healthspan^34, 35^. It appeared that resveratrol and STACs may use allosteric mechanisms to activate sirtuins and that they are also able to activate AMPK^35–38^. AMPK activation may support the neuroprotective effects of these compounds as AMPK is neuroprotective in *C. elegans*, cellular and mouse models of HD pathogenesis, with *daf-16*/FOXO-dependent effect in 128Q nematodes^23^. Here, we tested whether resveratrol neuroprotection may also be dependent on *bar-1*/ß-catenin, a FOXO co-factor^26^ that is important for FOXO3 neuroprotection in models of HD^18^, and the uncoupling protein gene *ucp-4/*UCP2, a *daf-16* transcriptional target that also has neuroprotective effects in models of HD^6^. Neuroprotection by resveratrol is lost in *bar-1* and *ucp-4* mutants (Fig. 2A), suggesting that resveratrol neuroprotection may engage DAF-16 signalling. The oxidative stress enzymes *sod-3*, a mitochondrial Fe/Mn superoxide dismutase, and the catalase *ctl-2* are two other well-characterized DAF-16 targets^39, 40^. The *sod-3* and *ctl-2* null mutants show no effect in 128Q animals (touch response in 24.6 ± 6% in 128Q*;sod-3/gk235* animals and 24.2 ± 3.7% in 128Q*;ctl-2*/*ok 1137*), but they are necessary for neuroprotection by resveratrol (Fig. 2B), further suggesting that resveratrol neuroprotection may engage DAF-16 signalling. Finally, consistent with our previously-reported observation for *daf-16*-dependent neuroprotection by GSK-3 inhibitors such as BIO^6^, lithium chloride (LiCl), a GSK-3 inhibitor with neuroprotective activity at low doses in the HD mice YAC128^22^, also protects 128Q nematodes from neuronal dysfunction in a *daf-16* dependent manner (Fig. 2C). Collectively, these data suggest that compounds using biological targets such as sirtuins, AMPK and ß-catenin (via GSK-3) all require *daf-16*/FOXO activity for neuroprotection against the HD protein.

### Isoquercitrin, a flavonoid with brain prodrug delivery potential, is neuroprotective in 128Q nematodes in a *daf-16*/FOXO, *sir2-1*/SIRT1 and *ucp-4*/UCP dependent manner

**Figure 3:**
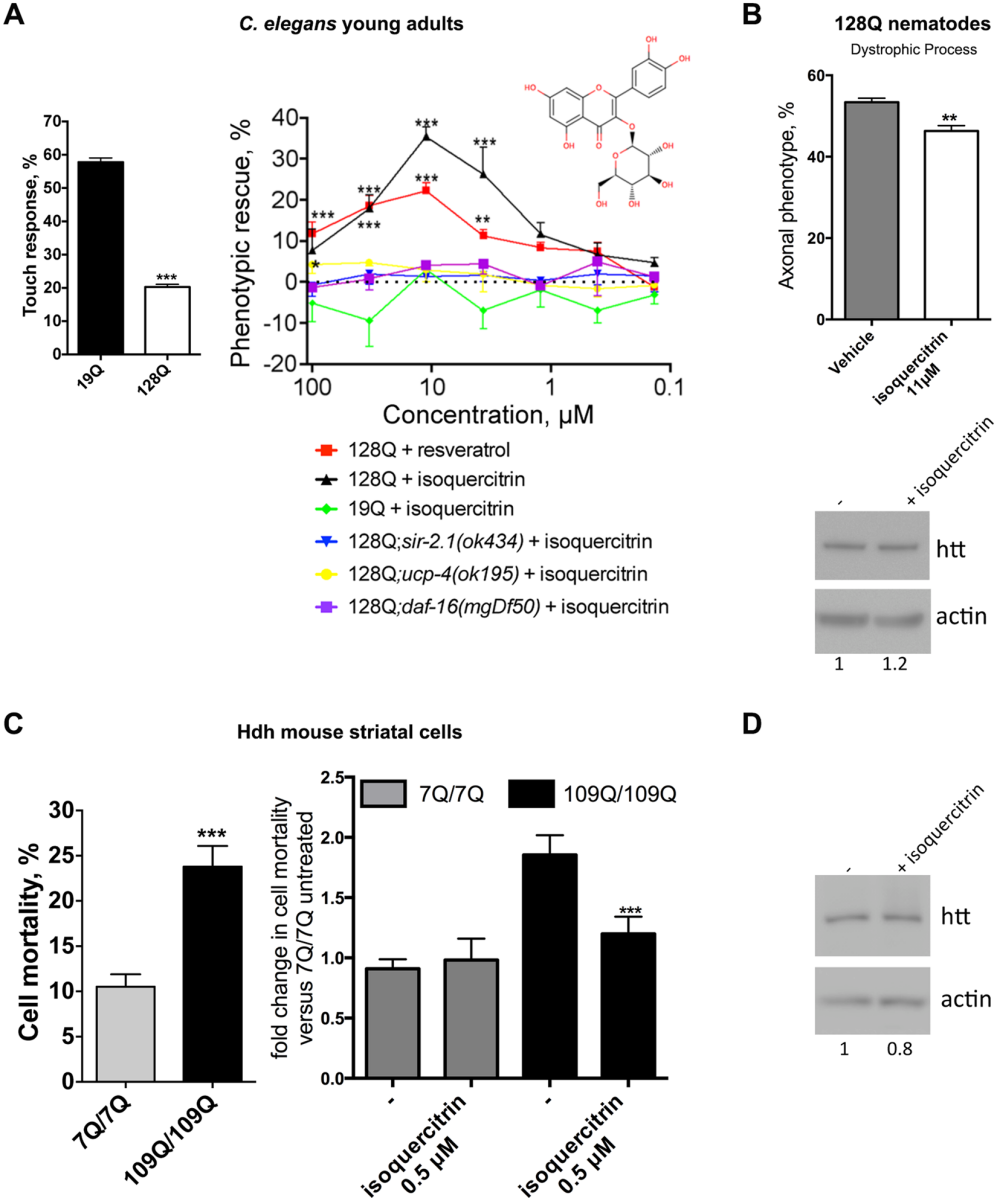
Protection of 128Q nematode neurons and mutant *Htt* mouse striatal cells by isoquercitrin. (**A**) The left panel show the level of touch response in 128Q and 19Q nematodes. The right panel shows that isoquercitrin suppresses the loss of touch response in 128Q nematodes, an effect that is lost in 128Q nematodes carrying a LOF mutation in *sir-2.1*, *daf-16* or *ucp-4*. The dose-response curve for resveratrol is shown for reference. Data are mean ± SEM for > 200 nematodes in each group and a total of at least 4 independent experiments. The percentage of phenotypic (touch sensitivity) rescue was calculated as ((test − control)/(100 − control)*100). (**A**) negative value for phenotypic rescue means aggravation of the phenotype. *P < 0.05, **P < 0.01 and ***P < 0.001 versus DMSO controls. ns: not significant. (**B**) The upper panel shows that isoquercitrin (11.1 µM) reduces the percentage of 128Q animals with axonal dystrophy. Data are mean ± SEM for > 200 nematodes in each group and a total of at least 4 independent experiments. **P < 0.01 versus DMSO controls. The lower panel shows that isoquercitrin (11.1 µM) has no effect on 128Q transgene expression (cropped representative western blot: the corresponding full-length blot is shown in Fig. S5). (**C**) The left panel shows cell vulnerability upon serum deprivation of mutant *versus* normal *Htt* mouse striatal cells. ***P < 0.001. The right panel shows that isoquercitrin (0.5 µM) reduces the vulnerability of 109Q/109Q cells to cell death upon serum deprivation, with no effect in 7Q/7Q cells. Data are mean ± SD for > 150 cells in each group as tested in at least three independent experiments. ***P < 0.001 versus DMSO controls. (**D**) Isoquercitrin (0.5 µM) has no effect on Htt expression in 109Q/109Q cells (cropped representative western blot: the corresponding full-length blot is shown in Fig. S6).

Targeting stress response pathways such as those regulated by FOXO and its co-factors has therapeutic potential for developing disease-modifying strategies in HD through the promotion of neuronal compensation. However, the repertoire of chemical compounds that may be used towards this end remains limited. For instance, resveratrol may protect neurons against HD by modulating the activity of stress-response pathways that converge onto FOXO such as Sir2^4^ and AMPK^23^ signalling, but the bioavailability and blood-brain barrier (BBB) properties of this compound are not favorable^41^. Having observed that *daf-16*/FOXO-dependent neuroprotection is a biological feature of several compound families, we searched for compounds that may promote the function of HD neurons in a FOXO-dependent manner while presenting good drug properties. To this end, we took advantage of the chemical diversity of flavonoids and screened a collection of 87 flavonoids carrying the resveratrol pharmacophore (Table S1) for modification of neuronal dysfunction (touch response at the tail) in 128Q nematodes. We identified 12 compounds showing dose-dependent (100–0.1 µM in culture media, which may translate into a 10- to 100-fold lower concentration into the animals) protection of neuronal function in 128Q nematodes and no detectable effect in 19Q nematodes. Hits were quercetin analogs and resveratrol analogs, all of them showing dependence on *sir-2.1*/SIRT1 for activity (Fig. S2; Fig. 3A). Among the most active hits are four quercetin analogs carrying a glucoside group. Interestingly, glycosyl derivatives have brain prodrug delivery potential^42^. We focused our attention on isoquercitrin (quercetin-3-O-glucoside) as this compound shows a better Rmax and EC50 compared to resveratrol and as the Rmax of isoquercitrin is very close to the maximally-achievable level of neuroprotection in the touch response assay (Fig. 3A). We found that, similarly to resveratrol, isoquercitrin is dependent on *daf-16*/FOXO and its transcriptional target *ucp-4*/UCP^6^ for neuroprotection in 128Q nematodes (Fig. 3A) with no detectable effect on the *HTT* transgene expression (Fig. 3B). Additionally, isoquercitrin specifically reduces the stress vulnerability of mutant *Htt* mouse striatal cells (Fig. 3C), with no effect on the expression levels of full length huntingtin (Fig. 3D). Collectively, these data suggest that medicinal chemistry studies can successfully identify families of neuroprotective compounds that may engage FOXO signalling while presenting a chemical structure that is suggestive of active drug properties.

### Integration of 128Q-nematode and Wormnet data provides a network-model and resource for studying neuronal compensation in Huntington’s disease

**Figure 4:**
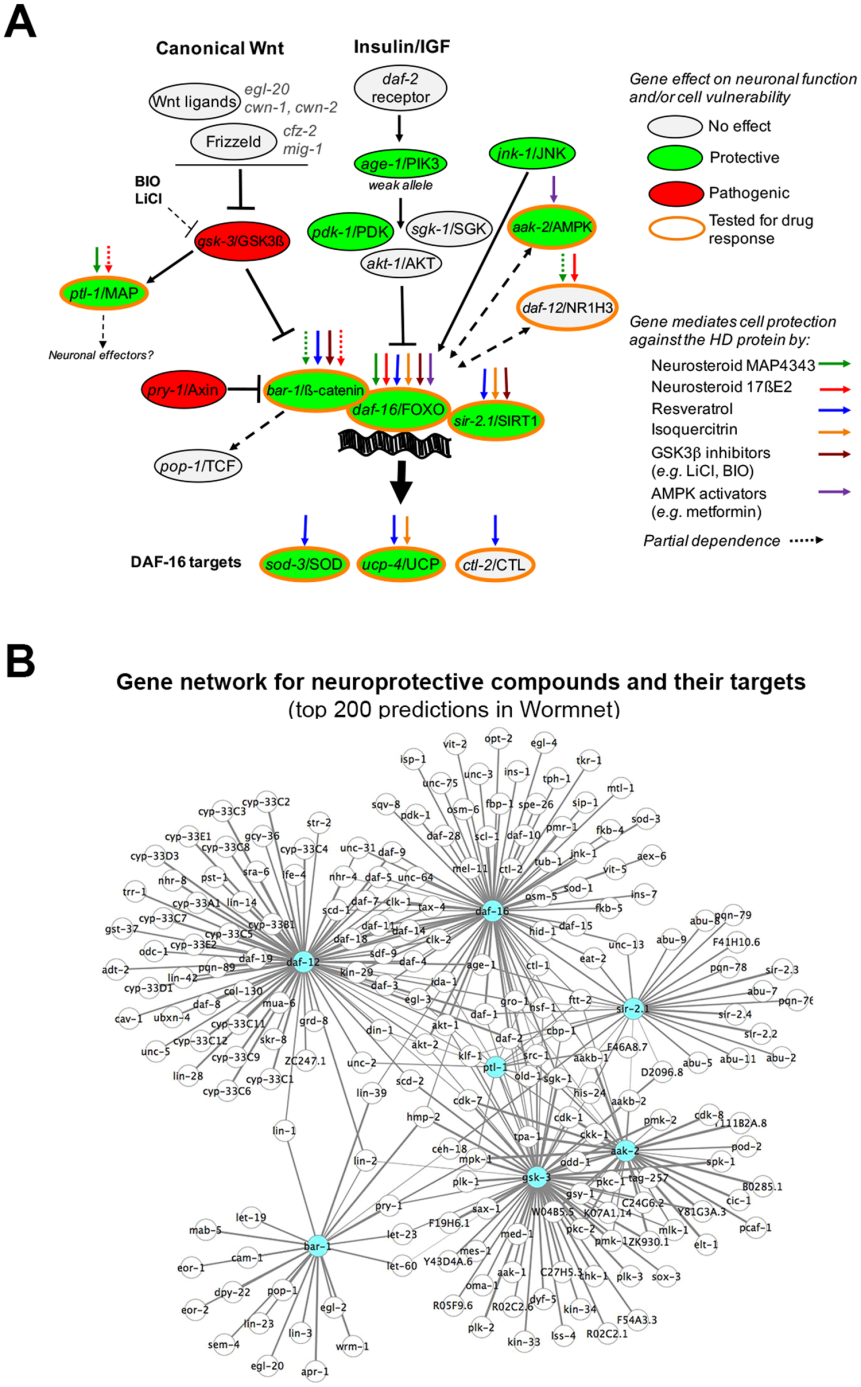
Current model for selective compound families to promote neuronal compensation in HD via a stress response system centered onto FOXO and its co-factors. This model is relevant to the early phases of mutant huntingtin cytoxicity, when neurons and cells are vulnerable but remain functional, and before they undergo degeneration. (**A**) Pathway layout that results from the synthesis of *C. elegans* (response to light touch mediated by PLM neurons) and/or mouse striatal cell (mortality induced by serum deprivation) data as reported herein for neurosteroids, resveratrol and isoquercitrin or elsewhere for resveratrol and metformin^6, 23^. Also included are *C. elegans* data on the effects of LOF mutants of genes in the canonical Wnt and insulin/IGF pathways (see Fig. S3). *Ptl-1*/MAP signaling is poorly understood, possibly involving intestinal *skn-1* for the regulation of stress response at the organismal level^99^. (**B**) Resource networks for investigating neuronal compensation mechanisms used by compounds shown in (**A**). The figure shows a sub-network of top 200 genes strongly predicted by Wormnet to interact with one or more of seed genes (shown in blue) including *sir-2.1*/SIRT1, *gsk-3/*GSK-3ß, *daf-16*/FOXO, *bar-1*/ß-catenin, *daf-12*/NR1H3, *aak-2*/AMPK and *ptl-1*/MAP2. This sub-network is selected from a network of 1267 genes (see Table S2) predicted by Wormnet^98^ to interact with *sir-2.1*/SIRT1, *gsk-3/*GSK-3ß, *daf-16*/FOXO, *bar-1*/ß-catenin, *daf-12*/NR1H3, *aak-2*/AMPK and *ptl-1*/MAP, the biological targets that may be used by the compounds tested herein. The top 200 predictions are based on the score for association to seed genes as provided in Wormnet. Edge thickness is proportional to the global-evidence score as provided in Wormnet for each gene-to-gene interaction in the graph. Table S2 shows whether inactivating gene neighbors may suppress or enhance neuronal dysfunction in 128Q nematodes as indicated by the overlap between the 1267 predictions and 662 genes previously identified to modify neuronal dysfunction upon RNAi knock-down in these animals^94^. The graph was generated using Cytoscape 3.3.0. The Cytoscape’s layout used for the graph is of the force-directed and spring-embedded type. This network is enriched for signalling pathways, cellular components and biological processes that are relevant to stress response and neuronal activity (see Results).

Our data indicate that several compound families can promote the function of 128Q nematode neurons in a *daf-16*/FOXO dependent manner, a common feature of neurosteroids (MAP4343, 17ßE2), flavonoids (resveratrol, isoquercitrin) and GSK3 inhibitors (LiCL, BIO) (Fig. 4A, Fig. S3). Isoquercitrin neuroprotection is dependent *ucp-4*/UCP, a DAF-16 target and resveratrol neuroprotection is dependent on DAF-16 targets *sod-3*/SOD, *ucp-4*/UCP and *ctl-2*/CTL (Fig. 4A), suggesting these two compounds may engage antioxidative response and mitochondrial efficiency. Additionally, we previously reported that AMPK activators such as metformin may also be dependent on daf-16/FOXO for neuroprotection in 128Q nematodes^23^ (Fig. 4A). Collectively, these data raise the possibility that multiple compound families might use a stress response network centered onto genes that function upstream or downstream to FOXO and its co-factors for promoting neuronal compensation in HD. To get further insight into this possibility, we used data from the probabilistic functional network Wormnet^43^ to identify the genes that may significantly interact with the targets engaged by MAP4343 (*ptl-1*/MAP), 17ßE2 (*daf-12*/NR1H3), resveratrol or isoquercitrin (*sir-2.1/*SIRT1, *daf-16*/FOXO), LiCl (*bar-1*/ß-catenin via *gsk-3*/GSK3) and resveratrol or metformin (*aak-2*/AMPK) for neuroprotection in 128Q nematodes. Wormnet analysis indicates these targets define a sub-network of 1267 genes (Table S2) having a node connectivity degree higher than occuring by chance (Wormnet: Area Under Receiver Operating Characteristic (AUROC) curve of 0.92 with *P* < 4.8 10^–22^). We next analyzed the biological significance of the gene nodes in this sub-network by using the STRING database. Interestingly, this network is enriched for stress response pathways such as the FOXO signalling (as expected), metabolic, ErB, mTor signalling, MAPK signalling, Wnt signalling and peroxysome pathways (KEGG pathways, *P* < 2.0 10^–5^). This network is also enriched in several biological processes (BP) that are involved in cell fate and metabolic regulation, among which several BPs that are important for stress response including cell cycle (*P* = 6.33 10^–15^), cell death (*P* = 1.33 10^–11^) and response to oxidative stress (*P* = 6.58 10^–9^). Finally, this network is enriched for several genes active in neuronal cell bodies (*P* = 3.6 10^–9^), axons (*P* = 2.9 10^–9^), dendrites (*P* = 5.84 10^–5^) and mitochondria (*P* = 7.91 10^–9^). This network contains interactors that are predicted to be strongly associated to seed genes (*sir-2.1*/SIRT1, *gsk-3/*GSK-3ß, *daf-16*/FOXO, *bar-1*/ß-catenin, *daf-12*/NR1H3, *aak-2*/AMPK and *ptl-1*/MAP2) as indicated by the Wormnet’s score for association with seed genes, a score that indicates the probability of an interactor to be in the same pathway as a seed gene. This is shown herein for the top 200 predictions (Table S2, yellow cells, score ranging from 4.22 to 1.87) (Fig. 4B). Interestingly, this sub-network is enriched for BPs (cell cycle: *P* = 0.00033; programmed cell death: *P* = 0.00407; response to stress: *P* = 8.09 10^–14^), Cellular Components (neuronal cell body: *P* = 4.25 10^–9^; axon: *P* = 2.98 10^–5^; dendrite: *P* = 2.29 10^–5^) and KEGG pathways (FOXO signalling, MAPK signalling, ErB, mTor signalling and Wnt signalling pathways, all of them with *P* < 3.0 10^–3^) that are similar to the ones for the network defined by 1257 interactors. Together, these data suggest that chemical compounds involving a *daf-16*/FOXO-interaction network for neuroprotection could constitute *bona fide* drugs for promoting stress reponse and neuronal compensation in HD.

## Discussion

The stress response and cell maintenance machinery contains several druggable targets that could be used for modifying the course of neurodegenerative diseases. However, the links between stress response targets and neuroprotective compounds remain poorly defined at the functional level. This is particularly true for compounds that may use FOXO-interaction networks^44^ to sustain the function of neurons expressing huntingtin, the HD protein. We thus explored the FOXO-dependent properties of neuroprotective compounds including neurosteroids such as MAP4343 and 17ßE2, flavonoids such as resveratrol, flavonoids that carry a resveratrol pharmacophore such as isoquercitrin, and GSK-3β inhibitors such as LiCl. The biological targets most-directly engaged by these compounds —*i.e*. nuclear hormone receptors, ß-catenin via GSK3ß, SIRT1 and AMPK— belong to pathways that signal onto FOXO or that cooperate with FOXO signalling for the regulation of stress response. Using *C. elegans* genetics, we found these compounds require *daf-16*/FOXO to promote neuronal function in adult 128Q nematodes. These compounds could thus use FOXO signalling, possibly engaging FOXO transcriptional targets that are important for stress response and neuronal compensation in HD. A FOXO-dependent neuroprotective activity of these compounds does not necessarily imply that they induce the nuclear translocation of DAF-16. These compounds indeed act on biological targets (*e.g*. GSK-3ß/ß-catenin, SIRT1, AMPK) that are known to finely tune FOXO activity in the nucleus. Given that the transcriptional activity of the FOXO3/ß-catenin complex is central to neuronal compensation in HD and that FOXO3 neuroprotection is altered during the early phases of the HD process^6, 9, 11, 18, 23^, these compounds —or similar compounds with better brain prodrug properties— could enable an efficient level of FOXO3 neuroprotection to be ensured in HD and the functional longevity of HD neurons to be prolonged. Interestingly, FOXO3 protects dopaminergic neurons from α-synuclein in models of Parkinson’s disease^9^. FOXO3 also protects motor neurons from mutant SOD1, mutant p150^glued^ and polyQ-expanded androgen receptor^45^. Additionally, *daf-16*/FOXO promote cell survival in response to aggregation of Aß(1–42), a peptide associated to Alzheimer’s disease^5^, and it mediates the protective effects of ethosuximide, an anti-epiletic drug with therapeutic potential for neuronal ceroid lipofuscinosis^46^. In so far, FOXO-interaction networks may provide a target space with therapeutic potential for several neurodegenerative diseases. Although FOXO factors protect against mild and manageable forms of cell stress, they may switch activity to triggering apoptosis as cells are facing un-manageable levels of stress and damage, which appears to be particularly true in post-mitotic neurons^47^. In so far, solely relying on models in which high levels of stress are acutely induced (*e.g*. overexpression of toxic disease protein fragments in cellular models) or models that recapitulate the most advanced stages of the disease process (*e.g. post-mortem* human brains) could be misleading on the role of FOXO factors in neurodegenerative diseases. While FOXO3 may promote apoptosis in dopaminergic neurons of the adult rat *substantia nigra pars compacta* subjected to acute oxidative stress, FOXO3 may protect these neurons against human α-synuclein expression^9^. FOXO factors also show protective effects in several models of neuronal dysfunction and cell vulnerability in HD^6, 18, 23, 48^. From a therapeutic prospective, this suggests that FOXO protection is relevant to the phases of neurodegenerative disease processes during which neurons retain the capacity to compensate for cellular stress and maintain function. Thus, the target space provided by FOXO-interaction networks may be most relevant to therapeutic intervention within a proper time window, *i.e*. before devastating brain cell loss. It is unclear whether targetting compensation systems such as FOXO-interaction networks might have therapeutic potential for treating the latest and most severe stages of HD. Nonetheless, targetting the early phases of HD in human individuals carrying the mutant *HTT* gene may become a realistic approach as biomarkers of pre-manifest and early-stage HD are being identified^49, 50^. Below we discuss how specific neuroprotective compounds may use this target space on a pathway engagement level.

Steroids have important roles in neuronal transmission, neuronal survival, myelination and cognitive function^51^. Additionally, steroids emerged as protective factors in the aging brain and peripheral neuropathy^52^. Noticeably, progesterone promotes neuroprotection and life span extension in the Wobbler mouse, a genetic model for motor neuron degeneration^53^. Our data emphasize steroids as a class of neuroprotective compounds that may act in a FOXO-dependent manner in neurodegenerative diseases such as HD. While dependence on *daf-16*/FOXO is a common feature for neuroprotection by MAP4343 and 17βE2, our data suggest they may use different activation mechanisms that involve either nuclear hormone receptors for response to 17ßE2 or MAPs for response to MAP4343. The latter mechanism is supported by the biochemical and biological evidence for MAP2 to be a neurosteroid receptor that binds MAP4343^19^. A role for MAPs to mediate MAP4343 neuroprotection is fully relevant to neuronal compensation in neurodegenerative disease as microtubule regulation is important for cell-survival/longevity^54^ and axonal transport^55^. Additionally, microtubule regulation is emerging as a common target in HD and Alzheimer’s disease^56^.

Our data also emphasize sirtuin modulators such as resveratrol^4^ and sirtinol^57^, GSK-3ß inhibitors such as BIO^6^ and polyphenols carrying a resveratrol-like pharmacophore such as isoquercitrin (this study) as neuroprotective compounds that may require FOXO factors for protection against the HD protein. Resveratrol appears to be well-tolerated at high doses in the mouse^34^, and it general activity may require key metabolic and stress response pathways whose components include SIRT1, PGC-1α and AMPK^34, 58–60^. Regarding its action against the HD protein, resveratrol is neuroprotective in nematode and mouse striatal cell models of HD pathogenesis^4^. Fisetin, a flavonoid with biological properties similar to resveratrol, is neuroprotective in multiple models of HD^61^. Additionally, resveratrol stands as one of the most potent molecules identified from greater than 1500 compounds evaluated in our *C. elegans* transgenics. Although resveratrol does not appear to show effects in the central nervous system of transgenic HD mice such as R6/2 mice^62^, these data call for the effects of compounds with a resveratrol pharmacophore to be revisited in mouse models of HD, notably in HD knock-in mice. In so far, an important aspect for the use of flavonoids like resveratrol in HD brain therapy is bioavailability and blood-brain-barrier (BBB) penetrance. Resveratrol has a short half life, poor bioavailability^63^, and while it may cross the BBB^64^, it remains unclear if a significant level of neuroprotection may be achieved in HD brains. Our results emphasize flavonoid-glucosides, notably quercetin-glucosides among which isoquercitrin, as potent neuroprotective compounds that require ß-catenin, Sir2 and UCPs for activity. Consistently, we previously found another neuroprotective flavonoid-glucoside, namely naringin, in the course of a drug screening effort that involved the screening of the NINDS Custom Collection in 128Q nematodes^65^. Quercetins may cross the BBB^66^, and quercetin protects against oxidative stress in *C. elegans* through DAF-16 activity^67^. Furthermore, flavonoid-glucosides have brain prodrug delivery potential. The interaction of the glycosyl with GLUT-1, a glucose transporter highly expressed in the BBB, may indeed help generate active compound concentrations in the brain^42^. Although some flavonoids such as quercetins might act promiscuously^68^, flavonol analogs such as isoquercitrin may constitute *bona fide* compounds that engage the stress response machinery (*e.g*. FOXO signalling) for sustaining the function of neurons facing proteotoxic stress (*e.g*. mutant HTT), making flavonoid analogs with a resveratrol pharmacophore a class of compounds worth investigating for their therapeutic potential in neurodegenerative diseases.

Studies have investigated whether compounds protecting from neurodegenerative disease associated proteins and polypeptides may act in a *daf-16* dependent manner. One compound, an iridoid, reduces aggregation of alpha-synuclein in a *daf-16* dependent manner in *C. elegans* transgenics that however express alpha-synuclein in body wall muscle cells^69^. Fluoxetine was reported to protect from Aß partly in a *daf-16* dependent manner in *C. elegans* transgenics that however express Aß in body wall muscles^70^. The data reported herein indicate that several compounds may protect in a *daf-16* dependent manner from neuronal dysfunction induced by polyQ-expanded exon-1 HTT expression. Lithium chloride and mythramycin reduce the death of ASH sensory neurons induced by polyQ-expanded N-terminal huntingtin expression in *C. elegans* and these effects are *daf-16* independent^71^, suggesting that *daf-16* dependent neuroprotection may primarily apply to the early stages of mutant HTT cytotoxicity, during neuronal dysfunction and before cell death.

To date, there is no efficient strategies for neuroprotection in neurodegenerative diseases such as HD. An innovative approach towards this end is the evaluation of RNAi strategies for lowering the expression of huntingtin^72, 73^. Another approach is to explore how cell replacement therapy could be effective in counteracting the clinical progression of HD^74^. Other approaches for disease modification could rely on the pharmacological stimulation of stress response and cellular maintenance systems for promoting neuronal compensation and, hopefully, delaying clinical onset of HD. Our data provide a model to further investigate how diverse compound families may implicate stress response pathways such as FOXO signaling into neuroprotective effects. The analysis of our data using Wormnet highlights a sub-network and model in which the routes for neuroprotection by individual compound families such as neurosteroids, flavonoids, GSK3 inhibitors and AMPK activators may be part of a larger regulatory system that controls stress response and that is enriched for neuronally-active genes. This model predicts there is tight integration of *sir-2.1*/SIRT1, *gsk-3/*GSK-3ß, *daf-16*/FOXO, *bar-1*/ß-catenin, *daf-12*/NR1H3, *aak-2*/AMPK and *ptl-1*/MAP2 signalling, highlighting a model in which the four families of compounds might be neuroprotective through common or partially-common stress response mechanisms. This prediction is supported by the published literature. 17βE2 binds to nuclear hormone receptors that are known to interact with FOXO factors. Interestingly, mammalian estrogen receptors physically interact with FOXO3 in suppressing cancer cell proliferation^75^ and DAF-12 physically interacts with DAF-16^76^, suggesting that the convergence of the nuclear hormone receptor and FOXO pathways that is exemplified at the network level is also implicated in 17βE2 neuroprotection. MAP4343 binds to *ptl-1*/MAP2^19^, and *ptl-1* is directly linked to *gsk-3* and indirectly linked to all of the seed genes used for Wormnet analysis (Fig. 4B). Noticeably, MAPs and FOXOs may share common biological functions as both *ptl-1* ^77, 78^ and *daf-16* ^79^ (as well as drosophila *FoxO* ^80^) may regulate axonal integrity during development and/or aging. Resveratrol is a *sir-2.1*/SIRT1 activating molecule^10, 34^ that may also activate AMPK^34, 81^, and both sirtuins and AMPK are longevity modulators that are well-known to modulate the activity of *daf-16*/FOXO^82–84^. GSK-3 inhibitors promote the activity of the GSK-3 target ß-catenin, a protein known to be a partner protein of DAF-16/FOXO in regulating oxidative stress^26, 85^ and protecting from the HD protein^6, 18^. Finally, AMPK activators such as metformin may protect neurons from the early phases of HD pathogenesis, when they are vulnerable yet functional, and before they undergo degeneration^11^. Regarding downstream effectors, some of which that appear in the network model (see Fig. 4B, *e.g. sod-3* for *daf-16, pop-1* for *bar-1*), it is important to consider that, while some FOXO targets are conserved across species and cell types, they can be dependent on the cellular context in which FOXO factors operate^86^. For instance, specific FOXO-target repertoires may be involved in the modulation of mouse neural stem cell homeostasis and synaptic activity as observed for FOXO3^87–89^ and FOXO6^90^. Thus, the pathways engaged by FOXO factors to promote biological processes such as organismal longevity are not necessarily the same as the ones engaged for modulating neuronal maintenance. Given the accumulating evidence for FOXO3 to have neuroprotective effects in several neurodegenerative diseases^5, 6, 9, 18, 46^, the network model shown herein provides a basis for future studies to investigate the FOXO targets and downstream mechanisms that could be engaged by selective compound families for prolonging neuronal function in HD.

In summary, our data indicate that stress response networks such as *daf-16*/FOXO-interaction networks may be used by several compound families for neuronal compensation in HD. This model has important therapeutic implications in HD and, perhaps, other neurodegenerative diseases as it provides a theoretical framework and unified rationale to search for disease-modifying strategies that tip the balance towards the prolongation of stress resistance and neuronal function in neurodegenerative disease.

## Materials and Methods

### C. elegans assays

We followed standard protocols for handling nematodes^91^. The *C. elegans* strains used were N2, ID1 (*exon1-HTT128Q::cfp;yfp*), ID245 (*exon1-HTT19Q::cfp;yfp*), ID447 (*exon1-HTT128Q*::*gfp*;*rrf-3(pk1426)*), ID448 (*exon1-HTT19Q*::gfp;*rrf-3(pk1426)*)*, bar-1(ga80)* EW15^92^, *lon-2(e678) daf-12(m20)* DR205, *daf-16(mgDf50)* GR1307^93^, *ptl-1(ok621)* RB809, *sod-3(gk235)* V433, *ctl-2*(*ok 1137)* VC754, *egl-20(n585)* MT1215, *cwn-1(ok546)* RB763, *cwn-2(ok895)* VC636, *cfz-2(ok1201)* RB1162, *pry-1(mu38)* CF491, *pop-1(q624)* JK2945 (partial LOF allele), *daf-2(e1370)* CB1370, *pdk-1(mg142)* GR1318 (dominant activating mutation), *pdk-1(sa680)* GT9609, *sgk-1(ok538)* VC345, *akt-1(mg144)* GR1310 (gain of function) and *akt-1(ok525)* RB759. All of these mutant strains carry a null allele unless otherwise indicated above.

RNAi assays for *gsk-3* were performed as previously described^94^ using the ID447 (128Q) as test strain and ID448 (19Q) as control strain. Bacterial clones from the *C. elegans* ORF-RNAi library were grown in 500 µl of LB culture medium supplemented with ampicillin (100 µg/ml) and tetracyclin (12.5 µg/ml), and they were induced by adding 10 µl of IPTG (12.5 mg/ml) 4 hours prior to incubation with nematodes. Synchronized L1 larvae were obtained by hypochlorite extraction using standard methods^91^ and they were grown at 20 °C onto Petri dishes until they reach the young adult stage. On the day of incubation in 96-well plates (D0), two young adult animals were incubated per well. Each of the wells contained 33.6 µl of bacterial culture and 16.6 µl of M9 media supplemented with cholesterol, ampicillin, tetracyclin, fungizone and IPTG. Incubation was performed at 20 °C during 4 days until the animals reached adulthood and produce late L4 larvae. To ensure appropriate feeding of the nematodes in the well, 16.6 µl of bacterial cultures expressing RNAi clones were added at Day 1. At Day 4, animals were transferred to agar plates and allowed to recover for 15 minutes and checked for lethality and developmental delay or other abnormalities. The two P0 animals (old and dark nematodes) initially incubated in the well were then manually removed from the plate and young adults (F1 progeny) were assayed for light touch response (see below).

Drug assays were performed blindly as previously described^4^. For drug screening, we used a collection of 87 highly pure compounds that carry the resveratrol pharmacophore (CHDI foundation, USA) (Table S1). In all drug assays, synchronized L1 larvae were obtained by hypochlorite extraction and incubated with drugs in 96-well plates in 50 µl S-Media with bacteria (OP50-1), 30 μg/ml streptomycin and 1% DMSO, at 23 °C until they reached young adulthood. Animals were then transferred to agar plates, allowed to recover and assayed for light touch response (see below).

In all types of experiments (genetic tests, RNAi tests, drug assays, drug screening), touch tests involved scoring for the response to light touch at the tail by using a fine hair. Touch tests were performed blindly by scoring 10 touches at the tail of the animal for a minimum of 200 animals per dose and at least four independent assays performed. Ordinarily, wild-type animals will respond by backing away from the touch. Loss of response to light touch at the tail is referred to as a tail Mec phenotype. The responses were recorded for every animal such that, for example, 3 responses out of 10 at the tail are given as 30% responsiveness, and the mean values for responsiveness were retained for comparison of nematode groups. To ensure the detection of highly active hits, compounds were tested at concentrations ranging from 0.1 µM to 100 µM in the culture medium, which may correspond to 10–100 times lower concentration in the animal. A minimum of 100–150 worms/test were scored per dose, and at least four independent assays performed. The percentage of phenotypic rescue was calculated as ((test − control)/(100 − control) * 100). A negative phenotypic rescue value means aggravation of the tail Mec phenotype. Rmax was calculated as the maximally achievable rescue and EC50 as the effective concentration at 50% rescue.

Scoring of PLM neuron morphology was performed as previously described^17^. ID1 animals were examined for PLM neuron morphological abnormalities and the presence of aggregates in neuronal processes. Briefly, 128Q nematodes treated with drug or vehicle were mounted on agar pads and immobilized using levamisole 20 mM, prior to examination on a 100 × objective of a Leica DMI8 microscope, equipped with fluorescence. A minimum of 100 animals of each type was scored for the presence of axonal swelling or aggregates in PLM neurons. Animals containing at least one swollen axon or one axon with aggregates were scored as positive.

Quantitative RT-PCR was performed on huntingtin-expressing transgenes in different worm mutants. RNA was isolated from young adult animals from stage-synchronized populations, by sonication, followed by extraction with a Qiagen Rneasy kit and DnaseI (ThermoFisher) treatment (as per the manufacturers’ protocol). Single strand cDNA synthesis was done using oligo(dT) priming and 200ng of total RNA with RevertAid First Strand cDNA Synthesis Kit (ThermoFisher). Quantitative PCR was performed using GoTaq Green Master Mix (Promega) with the Roche Light Cycler 480 and the oligonucleotides: 5′–3′, htt-f (CACTTGTCACTACTTTCTCAT), htt-r (GTAGTTCCCGTCATCTTTG), ama-1f (CCAACATCTCCAAGTTATGAAA), ama-1r (GATTGTATGTCGGCGAGGAT). Assays and data analysis were performed according to the manufacturer’s protocol (Roche). All samples were run at least in triplicate using *ama-1* as the calibrator gene with a dilution of 1/100 of cDNA. The amount of target, normalized to an endogenous reference (128Q) and relative to the calibrator (*ama-1*) was calculated using the 2^–∆∆CT^ method and statistical significance determined using paired *t* tests^95^.

Extraction of protein from whole worms and Western blotting was conducted using standard sonication methods^96^ and the following primary antibodies: GFP antiserum (Abcam, 1:5,000) and actin antibody (ThermoFisher, 1:5,000). Secondary antibodies used were as follows: goat–anti-rabbit IgG HRP-conjugated (Biorad, 1:10,000) and goat–anti-mouse IgG HRP-conjugated (Biorad, 1:10,000). Proteins were detected by using ECL + (ECL for actin) and evaluated by densitometry. At least three independent assays were performed. Signals were quantified using ImageJ.

### Mouse striatal cell assays

Cell vulnerability assays were performed as it follows. Low-passage normal (STHdh7Q) and mutant (STHdh109Q) *Htt* mouse striatal cells^20^ were cultured in 24-well plates and cell vulnerability assays performed as previously described using serum deprivation for cell death induction^97^. Serum deprivation was performed by replacing the medium with 1% FBS medium and with drug or vehicle. Cell vulnerability was scored 24 hours later upon DAPI staining and by counting pyknotic versus normal nuclei in DAPI-positive cells. This involved three independent experiments with 100 scored cells each, and assays were performed blindly. For Western blotting, proteins were extracted as previously described^97^, separated by SDS-PAGE, and analyzed by Western blotting using the following primary antibodies: mouse anti-HTT (4C8, Chemicon, 1:5,000) and mouse anti-actin (ThermoFisher, 1:5,000). The secondary antibody used was goat–anti-mouse IgG HRP-conjugated (Biorad, 1:10,000). Proteins were detected by using ECL + (ECL for actin) and evaluated by densitometry. At least three independent assays were performed. Signals were quantified using ImageJ.

### Network-based analysis

Sub-networks containing the seed genes of interest (*i.e. sir-2.1*/SIRT1, *gsk-3/*GSK-3ß, *daf-16*/FOXO, *bar-1*/ß-catenin, *daf-12*/NR1H3, *aak-2*/AMPK and *ptl-1*/MAP2) and their direct interactors were derived from the high coverage and probabilistic functional network Wormnet^98^. Network statistics (*i.e*. AUROC curve) were performed using the statistical tools provided in Wormnet. Biological content was analyzed using the STRING database (http://string-db.org/). Networks are represented using Cytoscape (http://www.cytoscape.org/).

### Statistics

Statistics of nematode data were performed using one-way ANOVA, with correction for multiple testing by Tukey’s Multiple Comparison Test. Statistics of striatal cell data and gene and protein expression data were performed using unpaired t tests. All experiments were repeated at least three times. All statistics were performed using GraphPad Prism. P < 0.05 was considered significant.

## Electronic supplementary material

Supplementary Information

Table S2
