# Supplementary material for: The stress response factor daf-16/FOXO is required for multiple compound families to prolong the function of neurons with Huntington’s disease
Source: Sci Rep. 2017 Jun 21;7:4014. doi: 10.1038/s41598-017-04256-w (PMC5479833; doi:10.1038/s41598-017-04256-w)
Supplement: Supplementary file 1 — Supplementary Information [file 41598_2017_4256_MOESM1_ESM.pdf]

Supplementary Information

The stress response factor *daf-16*/FOXO is required for multiple compound families to prolong the function of neurons with Huntington's disease

Francesca Farina[1,2], Emmanuel Lambert[1,2], Lucie Commeau[1,2,3], François-Xavier Lejeune[1,2], Nathalie Roudier[3], Cosima Fonte[3], J Alex Parker[1,2,†], Jacques Boddaert[1,2,4], Marc Verny[1,2,4], Etienne-Emile Baulieu[3,5] and Christian Neri[1,2]

[1]CNRS, UMR 8256, Laboratory of Neuronal Cell Biology & Pathology and University Hospital Department Fight Aging and Stress (DHU FAST), Paris, France, [2]Sorbonnes Universités, University Pierre and Marie Curie (UPMC) Univ Paris 06, Paris, France, [3]Inserm, UMR 788, 94276 Le Kremlin-Bicêtre, Cedex, France, [4]Department of Geriatrics, Pitié-Salpêtrière Hospital, Assistance Publique Hôpitaux de Paris (APHP), Paris, France, [5]MAPREG, 94276 Le Kremlin-Bicêtre, Cedex, France. Correspondence and requests for materials should be addressed to E.E.B. (email: etienne.baulieu@inserm.fr) or C.N. (email: christian.neri@inserm.fr).

†Current address: CRCHUM, Montréal, Canada and Department de Neurosciences, Faculté de médecine, Université de Montréal, Montréal, Canada

Supplementary Tables

Table S1. Effects of 87 compounds carrying the resveratrol pharmacophore on neuronal dysfunction in 128Q nematodes.

Compound ID		Effect on 128Q cytotoxicity			Comment
		Structure	Rmax, %	EC/ED50, µM	
1	NS218548	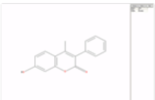	6.6 ± 27	n.a.	No protection
2	NS218514	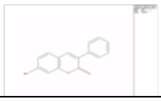	6.7 ± 3.4	n.a.	No protection
3	NS218469	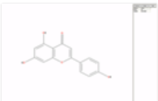	8.6 ± 4.6	n.a.	No protection
4	NS218472	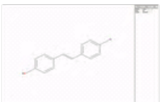	11.3 ± 4.8	n.a.	No protection
5	NS218557	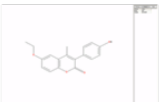	8.2 ± 3.3	n.a.	No protection
6	NS218554	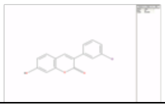	8.1 ± 8.8	n.a.	No protection
7	NS218552	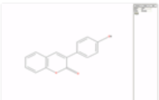	19.2 ± 5.1	2	Protection
8	NS218569	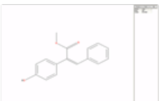	4.7 ± 4.5	n.a.	No protection
9	NS218570	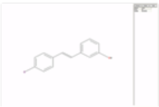	1.1 ± 5.5	n.a.	No protection
10	NS218546	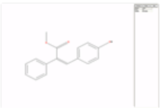	6.0 ± 5.6	n.a.	No protection

**11**	**NS218566**	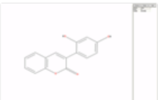	14.5 ± 3	n.a.	No protection
**12**	**NS218556**	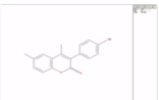	7.7 ± 1.7	n.a.	No protection
**13**	**NS218567**	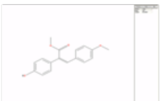	4 ± 1.4	n.a.	No protection
**14**	**NS218528**	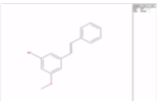	33 ± 7.1	20	Protection
**15**	**NS218568**	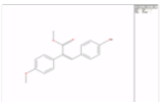	5.6 ± 6.4	n.a.	No protection
**16**	**NS218523**	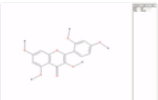	5.5 ± 0.5	n.a.	No protection
**17**	**NS218520**	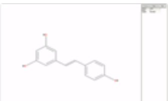	7.0 ± 1.1	n.a.	No protection
**18**	**NS218560**	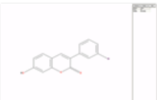	23.3 ± 6.8	20	Protection
**19**	**NS218508**	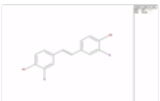	10.9 ± 0.2	n.a.	No protection
**20**	**NS218562**	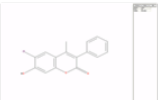	8.6 ± 3.1	n.a.	No protection
**21**	**NS218547**	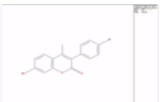	10.8 ± 0.5	n.a.	No protection
**22**	**NS218537**	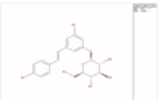	22.2 ± 6.3	10	Protection
**23**	**NS218553**	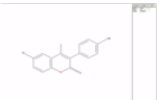	15.8 ± 17.6	n.a.	No protection

**24**	**NS218515**	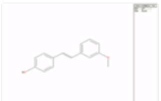	14.1 ± 5	n.a.	No protection
**25**	**NS218542**	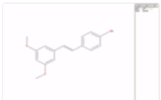	19.5 ± 9.4	5	Protection
**26**	**NS218555**	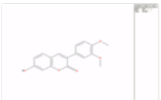	4.3 ± 7.2	n.a.	No protection
**27**	**NS218527**	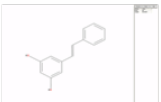	14.5 ± 1.42	n.a.	No protection
**28**	**NS218558**	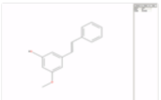	3.5 ± 3.1	n.a.	No protection
**29**	**NS218561**	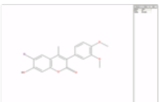	6.3 ± 10	n.a.	No protection
**30**	**NS218486**	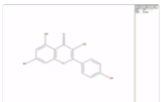	5.9 ± 3.5	n.a.	No protection
**31**	**NS218531**	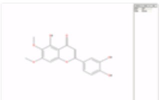	9.4 ± 10.3	n.a.	No protection
**32**	**NS218500**	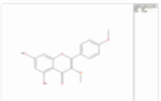	-0.1 ± 16.5	n.a.	No protection
**33**	**NS218517**	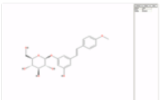	20.1 ± 2.5	n.a.	No protection
**34**	**NS218572**	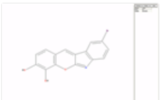	11.3 ± 0.3	n.a.	No protection
**35**	**NS218559**	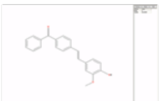	1.1 ±2.7	n.a.	No protection
**36**	**NS218504**	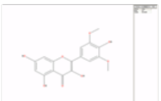	8.7 ± 3.7	n.a.	No protection

**37**	**NS218524**	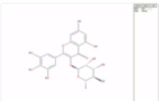	1 ± 0.9	n.a.	No protection
**38**	**NS218503**	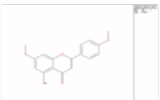	3.8 ± 6.5	n.a.	No protection
**39**	**NS218550**	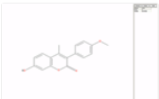	6.8 ± 1.7	n.a.	No protection
**40**	**NS218538**	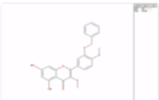	6.5 ±2	n.a.	No protection
**41**	**NS218498**	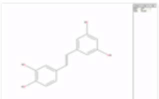	9.9 ± 8.2	n.a.	No protection
**42**	**NS218471**	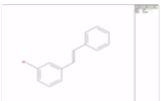	15.2 ± 1.7	n.a.	No protection
**43**	**NS218499**	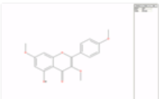	4.1 ± 0.3	n.a.	No protection
**44**	**NS218480**	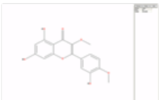	3 ± 7.3	n.a.	No protection
**45**	**NS218539**	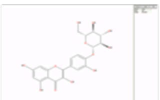	2 ± 12.1	n.a.	No protection
**46**	**NS218477**	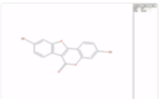	10.6 ±0.5	n.a.	No protection
**47**	**NS218485**	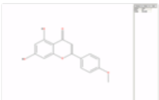	12.2 ± 3.6	n.a.	No protection
**48**	**NS218488**	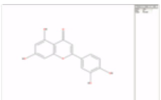	6.3 ± 1	n.a.	No protection
**49**	**NS218506**	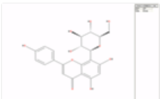	5.7 ± 1.3	n.a.	No protection

**50**	**NS218475**	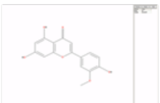	1.1 ± 3.3	n.a.	No protection
**51**	**NS218513**	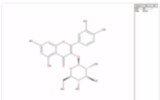	35.5 ± 2.3	2	Protection
**52**	**NS218549**	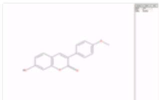	30.6 ± 4.5	10	Protection
**53**	**NS218525**	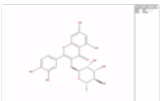	5.4 ± 2.5	n.a.	No protection
**54**	**NS218564**	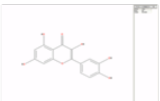	10.3 ± 4.6	n.a.	No protection
**55**	**NS218484**	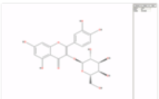	6.2 ± 1.6	n.a.	No protection
**56**	**NS218489**	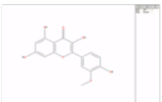	3.2 ± 1.3	n.a.	No protection
**57**	**NS218576**	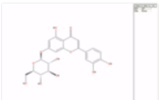	4.6 ± 2.6	n.a.	No protection
**58**	**NS218483**	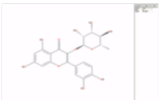	2.2 ± 9.8	n.a.	No protection
**59**	**NS218490**	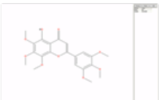	4.7 ± 3.5	n.a.	No protection
**60**	**NS218492**	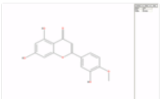	10.4 ± 1.4	n.a.	No protection
**61**	**NS218501**	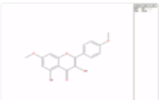	7.7 ± 1.1	n.a.	No protection
**62**	**NS218497**	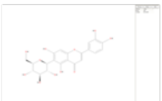	6.8 ± 3.1	n.a.	No protection

**63**	**NS218526**	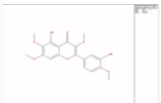	19.5 ± 3.8	5	Protection
**64**	**NS218487**	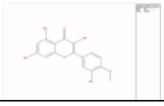	9 ± 2.3	n.a.	No protection
**65**	**NS218509**	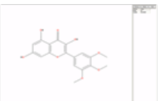	10 ± 4.1	n.a.	No protection
**66**	**NS218519**	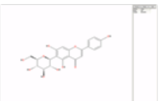	1 ± 0.1	n.a.	No protection
**67**	**NS218507**	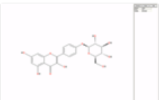	18.8 ± 5	6	Protection
**68**	**NS218518**	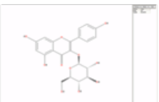	2.8 ± 1.4	n.a.	No protection
**69**	**NS218468**	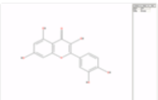	4.2 ± 1.4	n.a.	No protection
**70**	**NS218496**	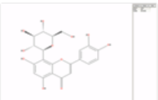	5.3 ± 1.5	n.a.	No protection
**71**	**NS218530**	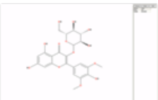	17.7 ± 4.6	2	Protection
**72**	**NS218536**	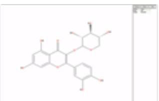	8.2 ± 0.8	n.a.	No protection
**73**	**NS218529**	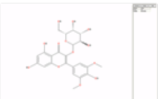	4.2 ± 1.6	n.a.	No protection
**74**	**NS218544**	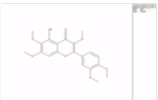	20.8 ± 4.9	1	Protection
**75**	**NS218474**	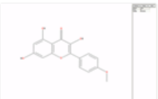	10.3 ± 7.3	n.a.	No protection

**76**	**NS218511**	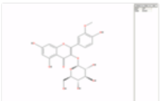	17.0 ± 3.8	3	Protection
**77**	**NS218467**	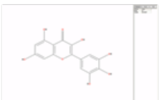	9.5 ± 2.5	n.a.	No protection
**78**	**NS218534**	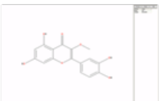	9.3 ± 7.5	n.a.	No protection
**79**	**NS218481**	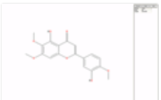	7.5 ± 3.7	n.a.	No protection
**80**	**NS218522**	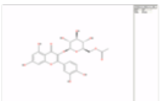	6.1 ± 2.3	n.a.	No protection
**81**	**NS218493**	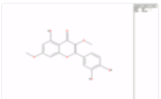	4.7 ± 1.6	n.a.	No protection
**82**	**NS218470**	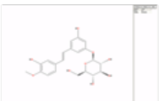	1.7 ± 4.5	n.a.	No protection
**83**	**NS218482**	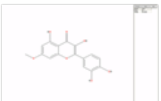	-0.2 ±6.2	n.a.	No protection
**84**	**NS218476**	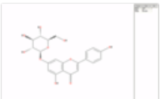	3.7 ± 1.3	n.a.	No protection
**85**	**NS218478**	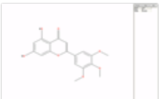	6.3 ± 1	n.a.	No protection
**86**	**NS218505**	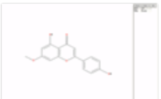	5.3 ± 7.8	n.a.	No protection
**87**	**NS218491**	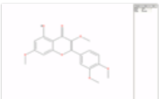	12.8 ± 5.1	n.a.	No protection

Nematodes were scored for sensitivity to posterior touch at various compound concentrations (100 - 0.1 μM). The percentage of phenotypic (touch sensitivity) rescue at the most effective compound concentration relative to untreated 128Q nematodes is indicated as the Rmax. The

EC50 is the half maximal effective concentration. The Rmax is the percentage of rescue at most effective concentration of drug relative to untreated control. The ED50 is the dose of a drug that is pharmacologically effective for 50% of the population. n.a.: not applicable. 'Protection' refers to compounds showing at least 2 active concentrations.

Table S2. Wormnet sub-network defined by seed genes *sir-2.1*/SIRT1, *gsk-3/*GSK-3ß, *daf-16*/FOXO, *bar-1*/ß-catenin, *daf-12*/NR1H3, *aak-2*/AMPK and *ptl-1*/MAP2. This sub-network contains 1267 genes. Yellow boxes indicate the top 200 predictions based on the Wormnet score for association with seed gene(s).

Table S2 is large: see content in the Table S2 file provided as a Supplementary dataset.

Supplementary Figures

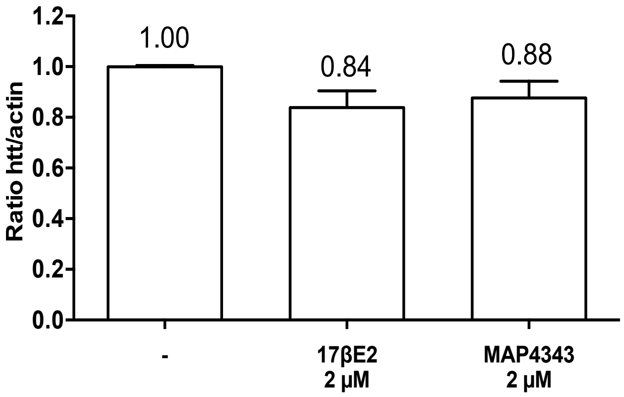

Figure S1. The expression of Htt is unchanged by 17ßE2 and MAP4343 treatment (2 µM) in mutant *htt* (109Q/109Q) mouse striatal cells. Data are mean±SE for a total of 3 independent experiments (Western blot analyzes).

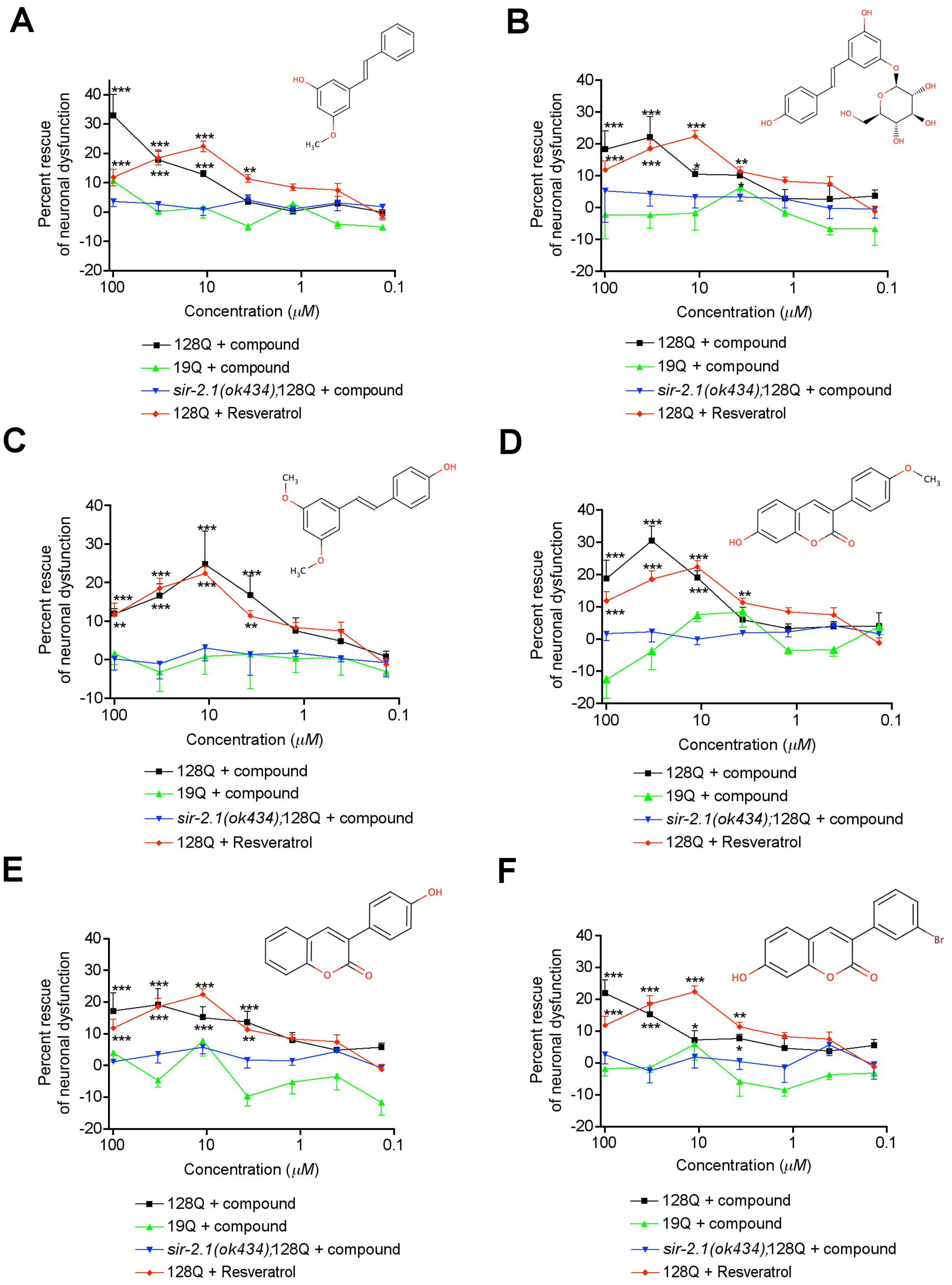
A
Percent rescue of neuronal dysfunction
Concentration (μM)
128Q + compound
19Q + compound
sir-2.1(ok434);128Q + compound
128Q + Resveratrol
B
C
D
E
F

G

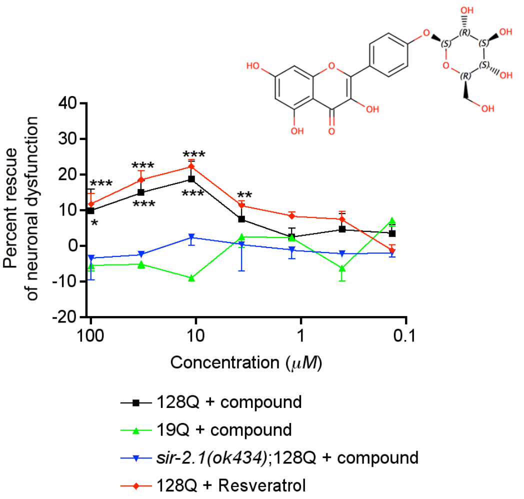
Percent rescue of neuronal dysfunction
Concentration (μM)
128Q + compound
19Q + compound
sir-2.1(ok434);128Q + compound
128Q + Resveratrol

H

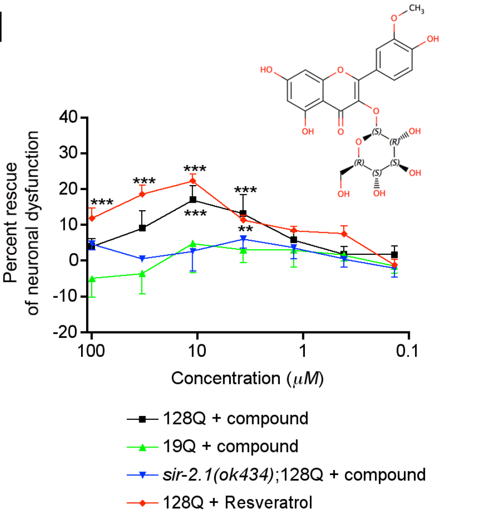
Percent rescue of neuronal dysfunction
Concentration (μM)
128Q + compound
19Q + compound
sir-2.1(ok434);128Q + compound
128Q + Resveratrol

I

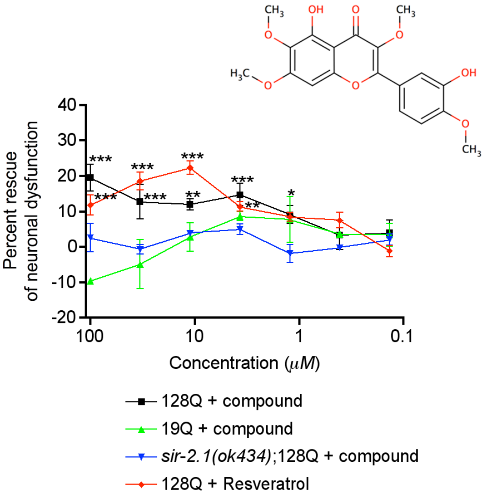
Percent rescue of neuronal dysfunction
Concentration (μM)
128Q + compound
19Q + compound
sir-2.1(ok434);128Q + compound
128Q + Resveratrol

J

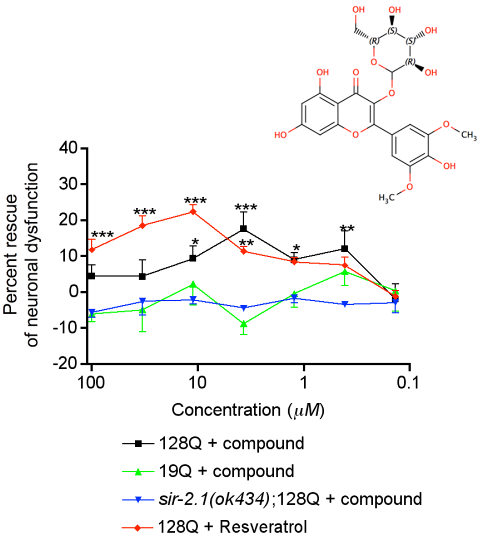
Percent rescue of neuronal dysfunction
Concentration (μM)
128Q + compound
19Q + compound
sir-2.1(ok434);128Q + compound
128Q + Resveratrol

K

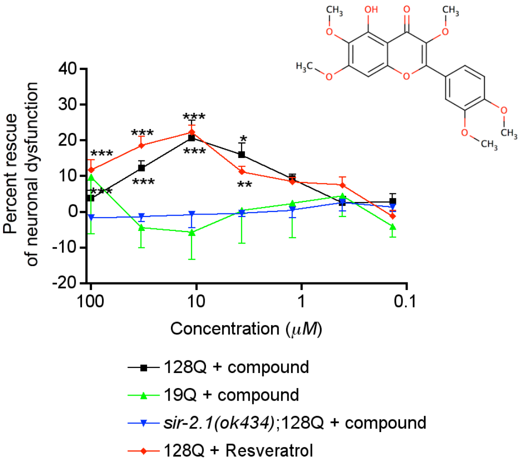
Percent rescue of neuronal dysfunction
Concentration (μM)
128Q + compound
19Q + compound
sir-2.1(ok434);128Q + compound
128Q + Resveratrol

Figure S2. Eleven compounds with the resveratrol pharmacophore that protect neuronal function in 128Q nematodes, and their structures. Hits fell into two chemical classes, namely resveratrol (**A, B, C**) and quercetin (**D** through **K**) analogs. The quercetin analog isoquercitrin, identified as the most potent hit, is shown in Figure 3. The resveratrol dose-response curve (red) is repeated for comparison. The percentage of phenotypic (touch sensitivity) rescue is calculated as ((test – control)/(100 – control)*100), and a negative value for phenotypic rescue means aggravation of the phenotype. The *P* values are shown only for amelioration of the tail mec phenotype. Data are mean±SEM for > 200 nematodes in each group and a total of at least 4 independent experiments. $^{*}P < 0.05$, $^{**}P < 0.01$ and $^{***}P < 0.001$ versus DMSO-treated 128Q animals.

A

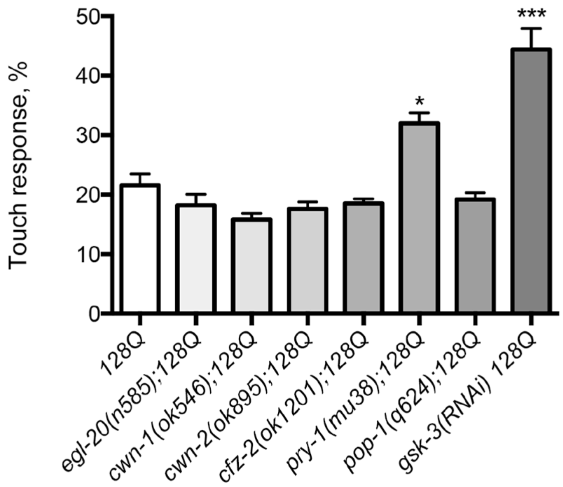

B

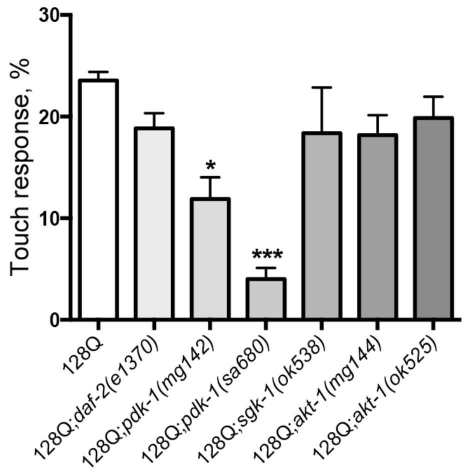

Figure S3. Effects of knocking out or reducing the activity of genes in the canonical Wnt and insulin/IGF1 pathways on touch response in 128Q nematodes. **(A)** Null allele of *pry-1* and *gsk-3* RNAi reduce the loss of touch response at the tail in 128Q animals. Data are mean±SEM for > 200 nematodes in each group and a total of at least 4 independent experiments. $*P < 0.05$ and $***P < 0.001$ versus 128Q animals. **(B)** LOF mutants of *pdk-1* aggravate the loss of touch response at the tail in 128Q animals. Data are mean±SEM for > 200 nematodes in each group and a total of at least 4 independent experiments. $*P < 0.05$ and $***P < 0.001$ versus 128Q animals. The genes tested (**A**, **B**) have no effect on touch response in 19Q nematodes.

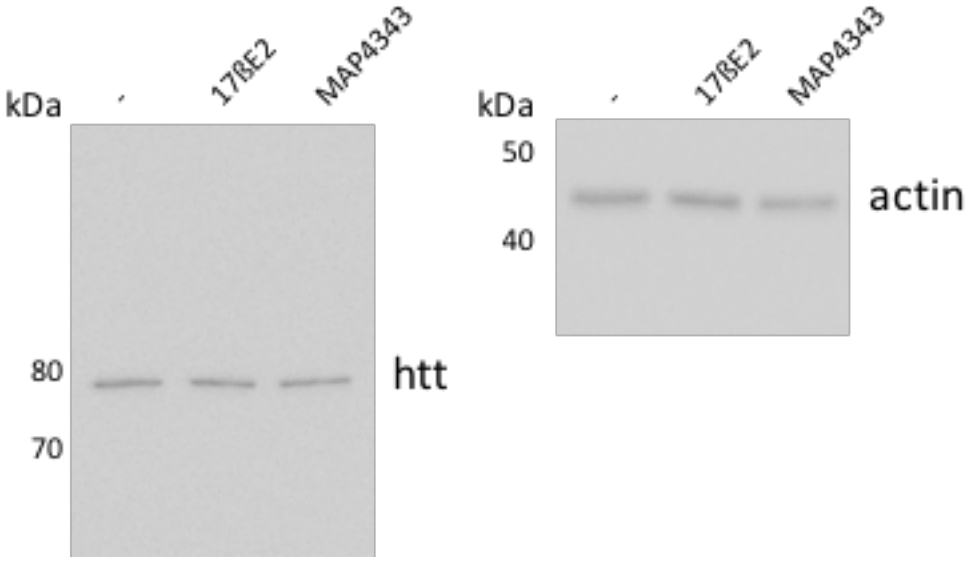

Figure S4. Full-length western blots corresponding to Fig. 1D (128Q nematodes).

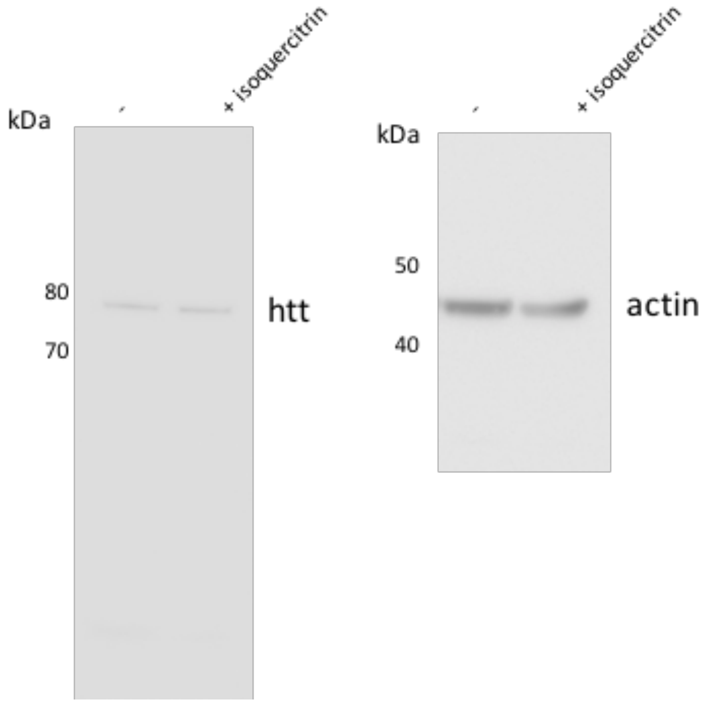

Figure S5. Full-length western blots corresponding to Fig. 3B (128Q nematodes).

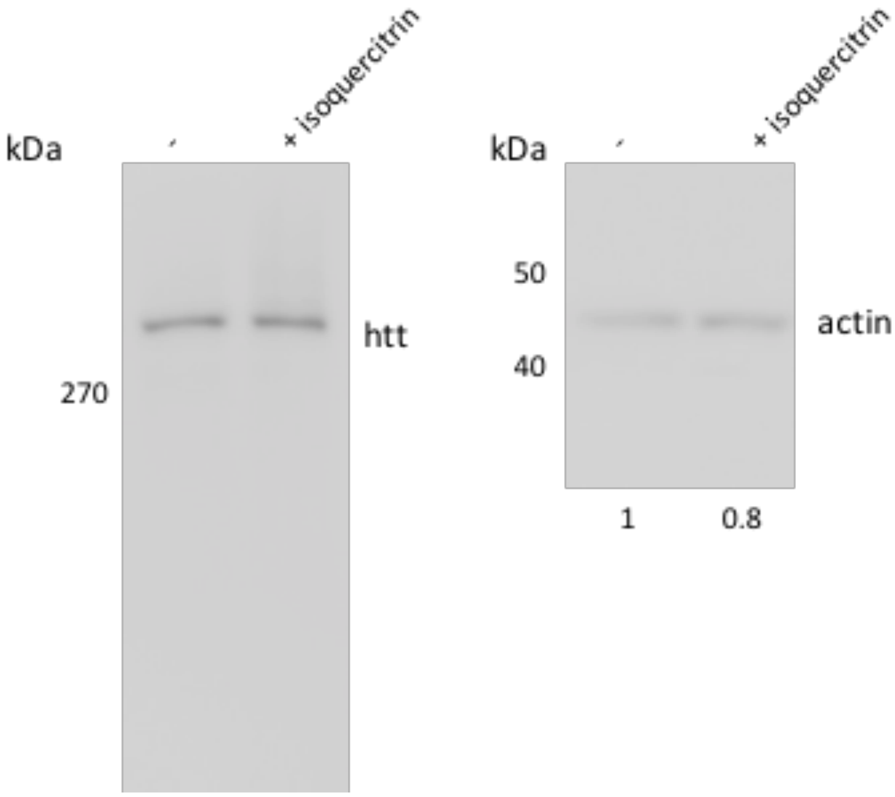

Figure S6. Full-length western blots corresponding to Fig. 3D (109Q/109Q mouse striatal cells).
